# Advanced airway interventions for paediatric cardiac arrest: updated systematic review and *meta*-analysis

**DOI:** 10.1016/j.resplu.2025.100963

**Published:** 2025-04-23

**Authors:** Jason Acworth, Jimena del Castillo, Elliott Acworth, Lokesh Tiwari, Jesus Lopez-Herce, Eric Lavonas, Laurie Morrison, Barnaby R. Scholefield, on behalf of the International Liaison Committee on Resuscitation Paediatric Life Support Task Force

**Affiliations:** a Emergency Department, Queensland Children’s Hospital, Brisbane, Australia; b Faculty of Medicine, University of Queensland, Australia; c Paediatric Intensive Care Department, Hospital General Universitario Gregorio Maranon de Madrid, Spain; d Intensive Care Unit, Gold Coast University Hospital, Southport, Australia; e Division of Pediatric Pulmonology and Intensive Care, Department of Paediatrics, All India Institute of Medical Sciences, Rishikesh, India; f Department of Emergency Medicine, Denver Health and Hospital Authority, Denver, USA; g Department of Emergency Medicine, University of Colorado School of Medicine, Aurora, USA; h Division of Emergency Medicine, Department of Medicine, University of Toronto, Toronto, Canada; i Rescu, Li Ka Shing Knowledge Institute, St. Michael’s Hospital, Toronto, Canada; j Department of Paediatric Critical Care, Hospital for Sick Children, Toronto and University of Toronto, Canada

**Keywords:** Paediatric, Cardiac arrest, Airway, Intubation, Ventilation, Supraglottic, Survival

## Abstract

**Background:**

Airway management is vital in paediatric resuscitation, especially since respiratory conditions are frequently the primary cause of paediatric cardiac arrest. Placement of an advanced airway device may facilitate more effective resuscitation than bag-mask ventilation but requires more skilled personnel and the time taken to perform the procedure may interfere with other vital components of resuscitation.

**Objectives:**

To assess the use of advanced airway interventions, tracheal intubation (TI) or supraglottic airway (SGA) placement, compared with bag mask ventilation (BMV) alone for resuscitation of children in cardiac arrest.

**Data sources:**

This was an update to a previous systematic review performed by ILCOR. A search of PubMed, EMBASE, and Cochrane Controlled Register of Trials (CENTRAL) was conducted for suitable studies published before 1 January 2025. This systematic review was registered as PROSPERO CRD42023482459.

**Study eligibility:**

Randomised controlled trials and non-randomised comparison studies involving airway interventions (BMV, TI, SGA) in infants and children (excluding newborn infants) in cardiac arrest in any setting were included.

**Study appraisal & synthesis:**

Investigators reviewed studies for relevance, extracted data, and assessed risk of bias using the RoB 2 and CLARITY frameworks. Critically important outcomes included survival to hospital discharge and survival with good neurological outcome.

**Results:**

We identified 20 suitable studies (13 from the original systematic review and 7 from the updated search), including 1 pseudorandomised clinical trial, 6 observational cohort studies using propensity matching, and 9 simple cohort studies suitable for *meta*-analysis. The majority of studies involved out-of-hospital cardiac arrest, with few studies exploring in-hospital cardiac arrest. The overall certainty of evidence was low to very low. For the critically important outcomes of survival to hospital discharge with good neurologic outcome and survival to hospital discharge, results showed no benefit from advanced airway interventions (TI or SGA) over BMV.

**Conclusions:**

There is currently no supporting evidence that an advanced airway (supraglottic airway or tracheal intubation) during CPR improves survival or survival with a good neurological outcome after paediatric cardiac arrest in any setting when compared with bag-mask ventilation.

Well-designed randomised trials are needed to address this important question.

## Introduction and background

Airway management is vital in paediatric resuscitation, especially since respiratory conditions are frequently the primary cause of paediatric cardiac arrest.^1^ Maintaining an open airway and delivering sustained effective ventilations using a bag-mask device can be difficult (even in skilled hands). Placement of an advanced airway device, such as a supraglottic airway or tracheal (endotracheal) intubation, may facilitate more effective resuscitation than the bag-mask ventilation (with or without an oropharyngeal or nasopharyngeal airway) but require more skilled personnel and the time taken to perform the procedure may interfere with other vital components of resuscitation e.g. chest compressions.^2^

Potential benefits of advanced airway device placement include more effective ventilation; prevention of aspiration of gastric contents (by preventing gastric distension and/or by mechanical protection of the upper airway); delivery of continuous as opposed to interrupted chest compressions; and more effective monitoring of CPR effectiveness and detection of return of circulation (ROSC) via end tidal carbon dioxide (EtCO2) measurement.^2^ Potential harms include incorrect tube placement or tube displacement leading to lack of lung ventilation or oxygenation; reduction in CPR quality secondary to prolonged interruption of CPR; and hyperventilation leading to respiratory alkalosis, reduced cerebral perfusion, or pneumothorax.^2^

A recent ILCOR Paediatric Life Support Task Force (PLS TF) systematic review published in 2019 ^3^ identified that neither tracheal intubation (TI) nor supraglottic airway (SGA) placement were associated with better outcomes than bag-mask ventilation (BMV) in paediatric cardiac arrest. This resulted in a change in treatment recommendation to a preference for the use of BMV rather than TI or SGA in the management of children during cardiac arrest in the out-of-hospital setting. Two further systematic reviews on this topic have been published since the ILCOR PLS TF systematic review.^3^ One examined pre-hospital paediatric airway management^4^ but included paediatric patients not in cardiac arrest. The other^5^ was limited to the prehospital setting only.

The current review represents an update to the previous systematic review^3^ to explore more recently published studies, in both out-of-hospital and in-hospital arrest settings, that could support or change current recommendations.

## Methods

This systematic review was commissioned by the International Liaison Committee on Resuscitation (ILCOR) Pediatric Life Support Task Force. The protocol for the systematic review was developed in accordance with the ILCOR framework and registered on PROSPERO (CRD42023482459) on 13 November 2023. The PROSPERO summary^6^ was updated on 8 April 2025 to improve consistency of definition of excluded studies (newborn rather than neonatal resuscitation) and to correct errors in the naming of a search database (PubMed rather than MEDLINE) and the study context section. The Preferred Reporting Items for Systematic Reviews and Meta-Analyses (PRISMA) checklist^7^ is included in the Supplemental Materials (S1).

### Eligibility criteria

Study characteristics used as criteria for eligibility are outlined in the PICOS question in Table 1.

**Table 1:** PICOS framework (Population, Intervention, Comparator, Outcome, Study Design).

**PICOS**	**Description**
**Population**	Infants and children who had received CPR after out-of-hospital or in-hospital cardiac arrest (excluding newborn children)
**Intervention**	Placement of an advanced airway device
**Comparison**	Compared with bag-mask ventilation (BMV) alone or with non–advanced airway interventions (Primary); or another advanced airway device (Secondary)
**Outcomes**	Survival to hospital discharge with good neurological outcome and survival to hospital discharge were ranked as critical outcomes. Return of spontaneous circulation (ROSC) was ranked as an important outcome.
**Study Design**	Randomised controlled trials (RCTs) and non-randomised studies (non-randomised controlled trials, interrupted time series, controlled before-and-after studies, cohort studies) were eligible for inclusion. Unpublished studies (e.g. conference abstracts, trial protocols) were excluded. All relevant publications in any language were included as long as there was an English abstract.

### Information sources

A professional librarian searched the PubMed, EMBASE and Cochrane Controlled Register of Trials (CENTRAL) databases using a peer-reviewed search strategy. As this was an update to a previously published ILCOR systematic review, all studies published from the most recent search date of the previous review (September 24, 2018) up until the updated search (August 15, 2023) were included and added to those from the original systematic review for analysis. The search was further updated on January 1, 2025. In addition, the authors reviewed international clinical trials registries, the bibliographies of published systematic reviews and of included studies, and review group members provided a list of known relevant studies.

### Search strategy

The main search terms included “cardiac arrest”, “pediatric”, “infant”, “child”, “intubation”, “supraglottic” and “laryngeal mask”. The complete search strategy is provided in the Supplemental Materials (S2).

### Study selection

Five authors (JA, JLH, EA, JdC, LT) independently reviewed the titles and abstracts of all articles identified in the search (2 authors per title). Copies were obtained of articles identified by either author. Two authors (JA, JdC) then reviewed the full text and identified articles meeting inclusion criteria. Disagreements were resolved by consensus.

### Data collection process

Two authors (JA, EA) separately extracted data from each included article to a Microsoft Excel spreadsheet. Discrepancies were resolved by consensus. When necessary, authors of included studies were contacted to clarify results.

### Data items

From each study, the number of children receiving each intervention (TI, SGA, and BMV) and the number of patients achieving successful resuscitation outcomes from each intervention were extracted. Patients in whom an intervention was attempted were included regardless of whether the intervention was successful (e.g. we analysed all children who received TI attempts, even if correct placement of the tube in the trachea did not occur). Propensity-matched data were used when available. In cases where case-level data could not be obtained from the publication or author, the study was not included in the *meta*-analysis and instead author-calculated adjusted odds ratios (aORs) for each interventions / outcomes were provided. If studies presented pooled data for all patients with advanced airway (AAW) attempts, combining outcomes achieved with TI and SGA, an attempt was made to contact the author for separate data. In keeping with the methods from the previous systematic review, if separate data could not be obtained, the study was categorised based on whether the majority of the AAW interventions were TI or SGA.

### Selection of outcomes

Prior to data extraction, the PLS Task Force identified two critically important outcomes: survival to hospital discharge with good neurologic outcome and survival to hospital discharge. To maintain consistency with the previous systematic review, when different studies measured specific outcomes at different time points, these were combined for analysis (e.g. results for survival with good neurologic outcome measured at discharge or 1 month later were combined). Good neurologic outcome was defined as cerebral performance score 1 – 2 or paediatric cerebral performance score 1 – 2.

### Risk of bias in individual studies

Risk of bias was assessed using the RoB 2 tool for clinical trials^8^ and the CLARITY framework for cohort studies.^9^

### Summary measures

All results are presented as Risk Ratios (RR) and absolute Risk Difference (aRD) and our assessment of statistical significance is based on the absolute risk reduction 95% confidence interval (CI 95%). A random effects model was chosen for *meta*-analysis to better account for study heterogeneity.

Summary effects were not reported if heterogeneity between studies was very high (I^2^ statistic greater than 75%).

### Synthesis of results

Results were compiled into a summary of findings table using the GRADEpro Guideline Development tool.^10^ Statistical calculations and Forest plot generation were performed using RevMan.^11^ Summary effect measures were generated for each study type (clinical trial, propensity-matched cohort study, or simple cohort study), but studies of differing design were not further combined.

### Certainty of evidence across studies

Certainty of evidence across studies was evaluated using the Grading of Recommendations Assessment, Development, and Evaluation (GRADE) framework.^12^ Inconsistency was considered serious if the I^2^ statistic was greater than 50%, and very serious if greater than 75%. Indirectness was considered serious if more than half the resuscitations in a study category were conducted prior to 2000 because of subsequent changes to standard resuscitation. Imprecision was considered serious if the CI for risk difference crossed two of the following points: +0.05 (appreciable benefit to the intervention), 0.00 (equipoise), and −0.05 (appreciable harm), and very serious if all three points were crossed.

### Additional analyses

The following subgroup analyses were prespecified at the time of protocol development: out-of-hospital cardiac arrest (OHCA), in-hospital cardiac arrest (IHCA), patients with cardiac arrest due to trauma compared to those with other causes of cardiac arrest, infants (age < 1 year), young children (1 – 12 years) and adolescents (13––18 years).

## Results

### Study selection

**Fig. 1:**
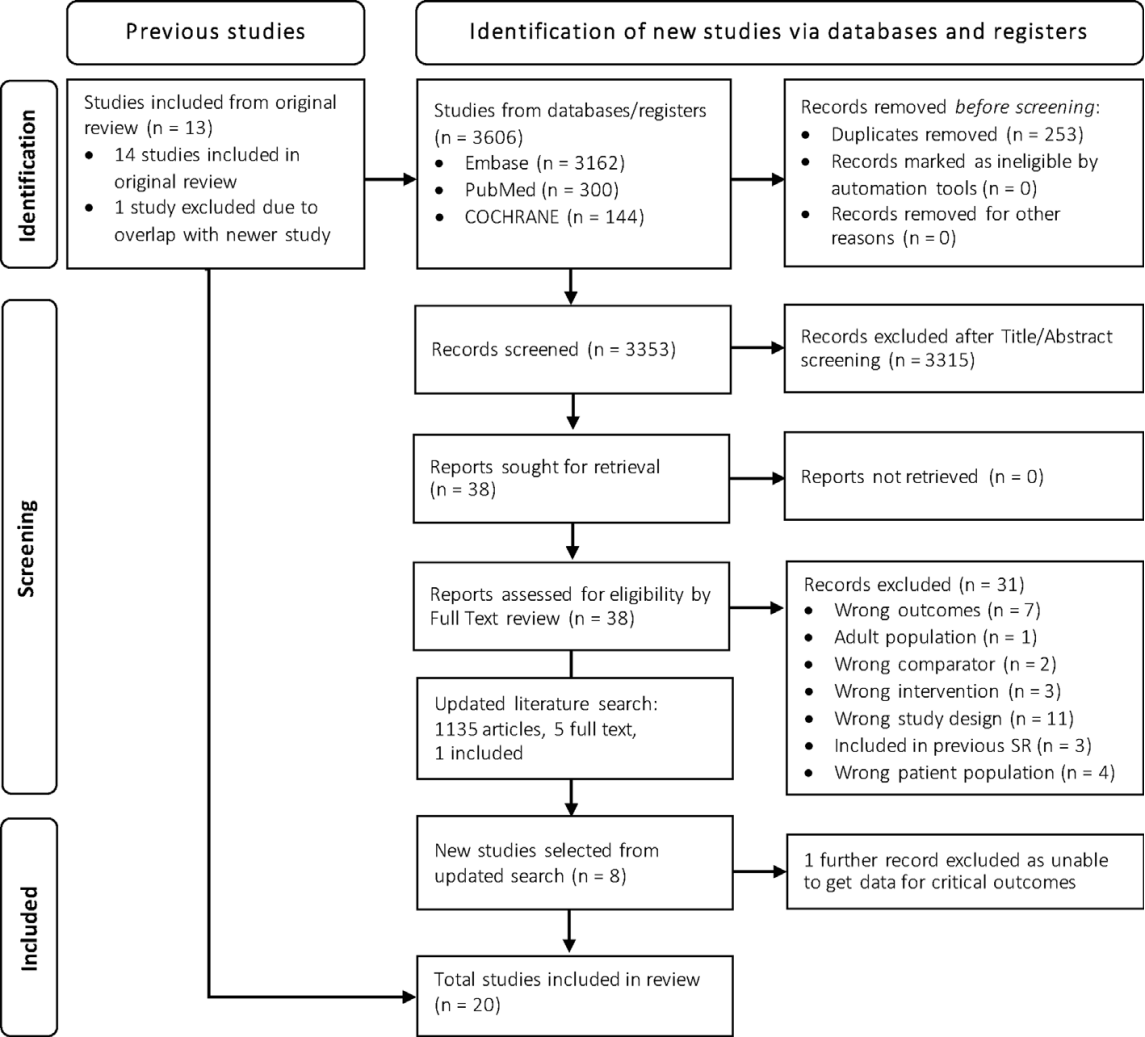
PRISMA Diagram for updated systematic review.

The initial search for this updated review was conducted on August 15, 2023, and an updated search up to January 1, 2025. Results were added to the 14 studies from the original systematic review for analysis. Results of each stage of the search are provided in Fig. 1. Taken together, the searches identified 7364 non-duplicate articles, of which 22 met inclusion criteria. One study^13^ that was included in the original systematic review^3^, ^14^ was excluded from this updated systematic review due to overlap with a newer study.^15^ Another study^16^ was excluded as outcome data was only available for ROSC rather than the determined critical outcomes.

### Study characteristics

Twenty studies were included in the systematic review. Only 1 study provided clinical trial data.^17^ Six studies provided propensity-adjusted cohort data suitable for *meta*-analysis.^15^, ^18^, ^19^, ^20^, ^21^, ^22^ Nine other studies provided retrospective cohort data amenable to *meta*-analysis.^13^, ^23^, ^24^, ^25^, ^26^, ^27^, ^28^, ^29^ Five studies provided retrospective cohort data in adjusted form only, not amenable to *meta*-analysis.^14^, ^16^, ^30^, ^31^, ^32^ Characteristics of these studies are described in Table 2**.**

**Table 2:** Characteristics of included studies.

**Study**	**Years conducted**	**Setting**	**Location**	**Study Participants**	**Interventions Compared**	**Outcomes Assessed**
**Pseudo-randomised controlled clinical trial**						
Gausche 2000	1994–1997	OHCA	USA	n = 591 paediatric (aged ≤ 12 years) with OHCA. 301 received TI, 290 received BMV.	TI v BMV	SGNF, SHD
**Cohort Studies: Propensity-Matched**						
Andersen 2016	2000 – 2014	IHCA	USA	n = 2294 paediatric (aged < 18 years) with IHCA. 1555 received TI, 649 no TI. n = 2270 in propensity matched cohort.	TI v no AAM	SGNF, SHD, ROSC (sustained 20 min)
Fukuda 2020	2011–2017	OHCA	Japan	n = 967 eligible paediatric (aged 1–17 years) OHCA patients who received prehospital AAM by EMS personnel. 113 (11.7%) received TI, and 854 (88.3%) received SGA. 113 patients receiving TI were propensity-matched with 113 patients receiving SGA insertion.	TI v SGA	SGNF (assessed at 1 month), SHD (1 month survival), ROSC (pre-hospital ROSC)
Hansen 2017	2013–2015	OHCA	USA	n = 1724 paediatric (aged < 18 years) with OHCA. 727 received TI, 215 received SGA, 781 received BMV only.	TI v SGA v BMV	SGNF, SHD, SHA, ROSC (sustained 20 min)
Ohashi-Fukuda 2017	2011 – 2012	OHCA	Japan	n = 2157 paediatric (aged 1–17 years) with OHCA. 33 received TI, 332 received SGA, 1792 received BMV only. In the propensity matched cohort, 31 received TI, 315 received SGA, 346 received BMV only.	TI v SGA v BMV	SGNF (assessed at 1 month), SHD (1 month survival), ROSC (pre-hospital ROSC)
Okubo 2019	2014–2016	OHCA	Japan	n = 3801 children (<18 years) with OHCA. 63 received TI, 418 received SGA, 3320 received no AAM.	TI v SGA v no AAM	31-day survival, SGNF
Tham 2022	2009 – 2018	OHCA	Japan, Korea, Malaysia, Singapore, Taiwan, Thailand, UAE, India, China, Philippines, Vietnam	n = 3131 children (<18 years) with OHCA. 81 received TI, 371 received SGA, 2679 received BMV.	TI v SGA v BMV	SHD (1 month survival), SGNF
**Cohort Studies: Not Propensity-Matched**						
Abe 2012	2005 – 2008	OHCA	Japan	n = 3189 infants (aged < 1 years) with OHCA.	TI v SGA v BMV	SHD (1-month survival)
Aijian 1989	1984 – 1987	OCHA	USA	n = 63 paediatric (aged < 19 years) with OHCA. 28 received TI attempts, 14 received BMV only.	TI v no AAM	SHD, SHA
Deasy 2010	1999 – 2007	OHCA	Australia	n = 193 paediatric (aged < 16 years) with OHCA. 167 received TI attempts, 26 received BMV only.	TI v no AAM	SHD (1 month survival)
del Castillo 2015	2007 – 2009	IHCA	Argentina, Brazil, Columbia, Chile, Ecuador Honduras, Italy, Paraguay, Portugal, Spain	n = 502 paediatric (aged 1 m to 18y) with IHCA.	TI v no AAM	SGNF (assessed at 1 year)
Guay 2004	1983 – 1987	IHCA	Canada	n = 145 paediatric (aged ≤ 21 years) with IHCA. 90 received TI attempts, 55 no TI attempt.	TI v no AAM	SHD (1 year survival)
Handley 2021	2006–2018	IHCA	USA	n = 3521 infants with IHCA. 66% (n = 2341) of infants had a CPR event in the NICU and 34% (n = 1180) of infants were in the PICU. 1159 (33%) had TI, 2362 (67%) had no AAM. Newborns excluded.	TI v no AAM	ROSC, 24-hr Mortality, SHD
Hansen 2020	2016–2018	OHCA	USA	n = 154 children (<18 years) with EMS-treated non-traumatic OHCA (chest compressions by an EMS provider or a defibrillator shock prior to EMS arrival with an AED)	TI v SGA v no AAM	SHD
Pitetti 2002	1986 – 1999	OHCA	USA	n = 189 paediatric (aged ≤ 18 years) with OHCA. 150 received TI attempts, 39 no TI attempt.	TI v no AAM	SHD, SHA
Sirbaugh 1999	1992 – 1995	OHCA	USA	n = 255 paediatric (aged ≤ 18 years) with OHCA. 222 TI, 26 no AAM, 7 already had tracheostomy.	TI v no AAM	SGNF, SHD, ROSC (pre-hospital ROSC)
**Cohort Studies: Not Propensity-Matched, Not Amenable to Meta-Analysis**						
Cheng 2021	2015–2019	OHCA	Taiwan	n = 124 paediatric (age ≤ 20 years) OHCA patients from the emergency medical service (EMS) database of Kaohsiung City	TI and SGA combined v no AAM	SHD, SGNF
Fink 2016	2007 – 2012	OHCA	USA	n = 1738 children (0–19 years) with OHCA from ROC-Epistry. 900 attempted TI, 82 attempted SGA, 1346 received BMV.	TI and SGA combined (92% TI) v no AAM	SHD, SHA
Katzenschlager 2023	2007–2021	OHCA	Germany	n = 1579 children (<18 years) with OHCA. 1070 received TI, 320 received SGA, 67 BMV only.	TI and SGA combined (77% TI) v no AAM	ROSC
LeBastard 2021	2011–2018	OHCA	France	n = 1579 children (<18 years) with OHCA. 1355 received TI, 16 received SGA, 208 received BMV.	TI v (SGA or BMV)	30-day survival, ROSC, SGNF
Tijssen 2015	2005 – 2012	OHCA	Canada, USA	n = 2244 children (0–19 years) with OHCA from ROC-Epistry. 1449 attempted TI, 109 attempted SGA, 2105 received BMV.	TI and SGA combined (93% TI) v no AAM	SHD

**AAM:** Advanced Airway Management; **BMV**: bag mask ventilation; **EMS**: Emergency Medical Services; **IHCA**: in-hospital cardiac arrest; **NICU**: Neonatal Intensive Care Unit; **PICU**: Paediatric Intensive Care Unit; **OHCA**: out-of-hospital cardiac arrest; **ROSC**: return of spontaneous circulation; **SGA**: supraglottic airway; **SGNF**: survival with good neurologic function; **SHA**: survival to hospital admission; **SHD**: survival to hospital discharge; **TI**: tracheal intubation

### Risk of bias within studies

The risk of bias in each study is presented in the Supplemental Materials (S3)**.** One clinical trial was identified as having low risk of bias.^17^ Adherence to the intended intervention was transparent and high, though some cross-over was reported.

Seven retrospective cohort studies using propensity-matching methods were judged to have serious risk of bias.^15^, ^18^, ^19^, ^20^, ^21^ These studies each utilised rigorously collected registry data and applied propensity-matching methods to attempt to account for the likelihood that TI would be attempted, or not attempted, for a given patient. However, none of these studies were able to distinguish patients who did not have attempts at TI from those in whom TI was attempted unsuccessfully. In addition, as in all propensity-controlled studies, it is impossible to estimate how effectively propensity methods reduced bias.

Twelve retrospective cohort studies that did not use propensity matching and met inclusion criteria were judged to have very serious risk of bias, primarily because it was impossible to determine why some patients were intubated and some were not.^13^, ^14^, ^23^, ^24^, ^25^, ^26^, ^27^, ^28^, ^29^, ^30^ Nine of these studies included raw data amenable to *meta*-analysis, either in the published manuscript or provided by the author.^13^, ^23^, ^24^, ^25^, ^26^, ^27^, ^28^, ^29^.

### Synthesis of results

**Fig. 2:**
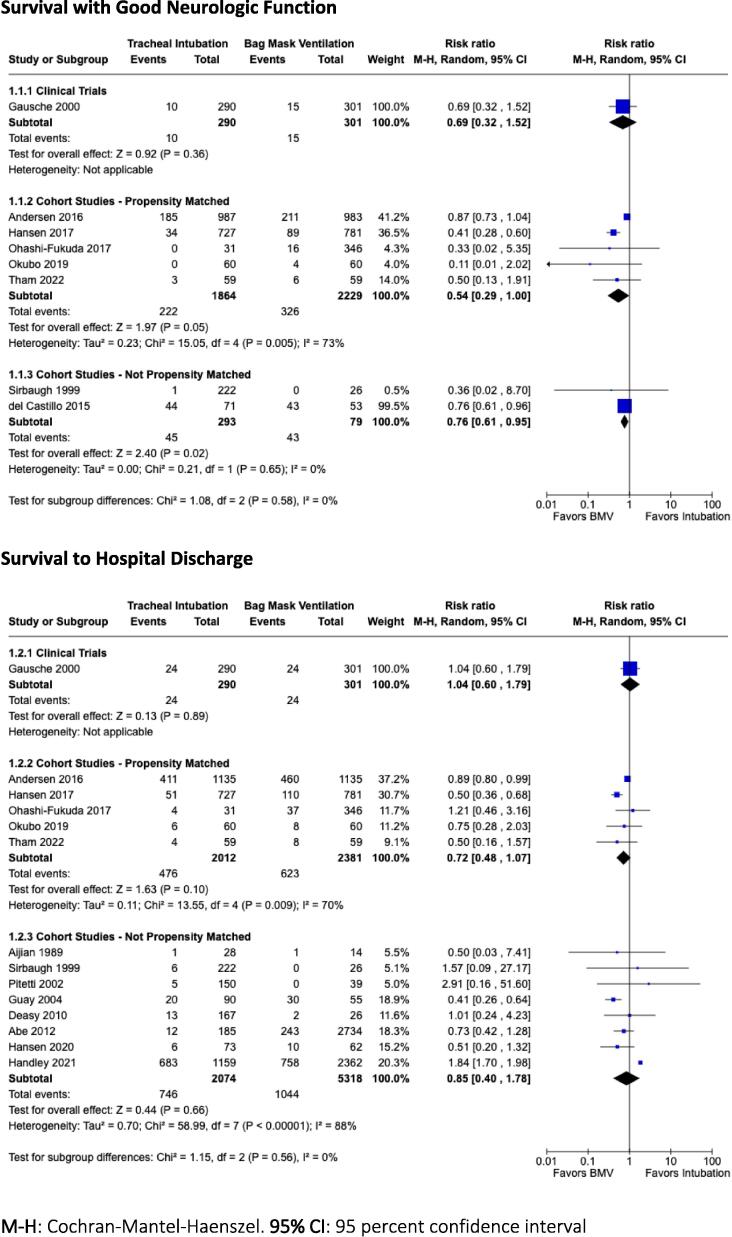
Forest Plots comparing Tracheal Intubation to Bag Mask Ventilation for infants and children in cardiac arrest for the critical outcomes of Survival with Good Neurologic Function and Survival to Hospital Discharge. **M−H**: Cochran-Mantel-Haenszel. **95% CI**: 95 percent confidence interval.

**Fig. 3:**
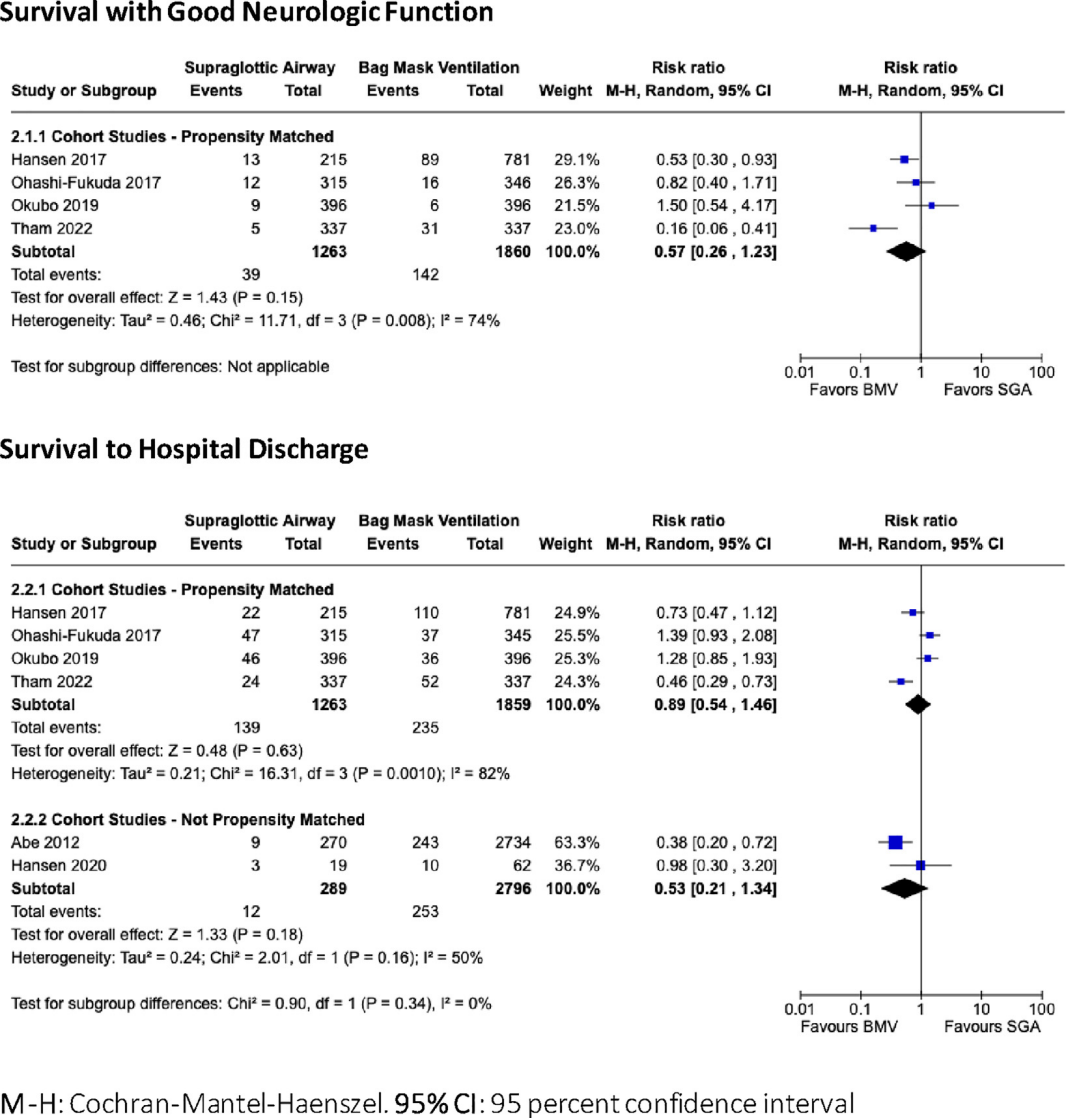
Forest Plots comparing Supraglottic Airway Placement to Bag Mask Ventilation for infants and children in cardiac arrest for the critical outcomes of Survival with Good Neurologic Function and Survival to Hospital Discharge. **M−H**: Cochran-Mantel-Haenszel. **95% CI**: 95 percent confidence interval.

**Fig. 4:**
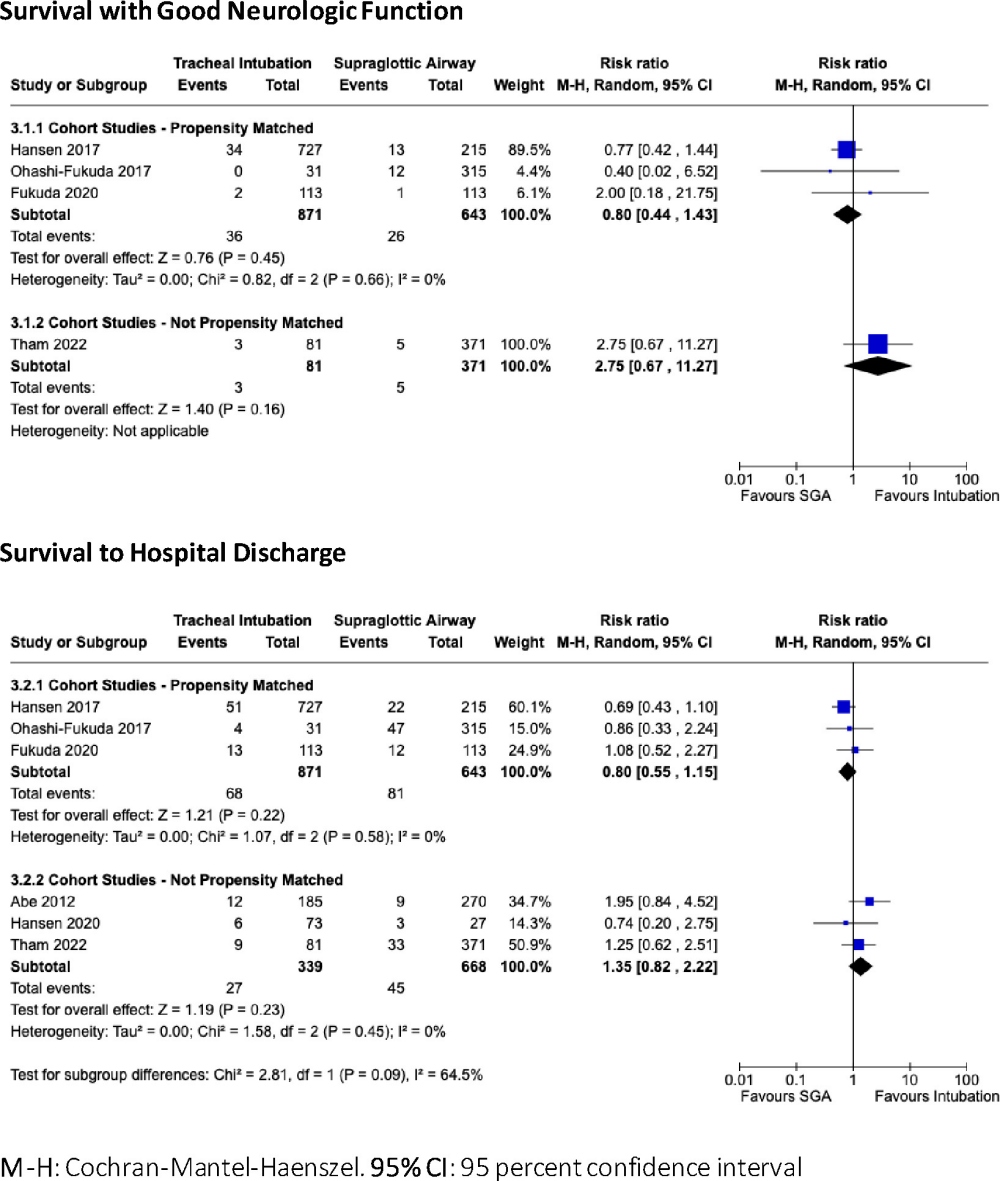
Forest Plots comparing Tracheal Intubation to Supraglottic Airway Placement for infants and children in cardiac arrest for the critical outcomes of Survival with Good Neurologic Function and Survival to Hospital Discharge. **M−H**: Cochran-Mantel-Haenszel. **95% CI**: 95 percent confidence interval.

GRADE Summary of Findings for the critical outcome comparisons are presented in Table 3. Forest plots for the critical outcome comparisons are presented in Fig. 2, Fig. 3, Fig. 4.

**Table 3:** Summary of Findings for Critical Study Outcomes.

**Outcomes**	**Participants (Studies), n**	**Certainty of Evidence (GRADE)**	**RR (95% CI)**	**Absolute Risk with Comparator**	**ARD with Intervention**
TI (I) *vs* BMV (C)					
Survival with favourable neurologic outcome	591 (1 RCT)	Low	RR 0.69 (0.32 to 1.52)	50/1000	15 fewer per 1,000 (from 34 fewer to 26 more)
	4093 (5 propensity-matched observational)	Very low	RR 0.54 (0.29 to 1.00)	146/1000	67 fewer per 1,000 (from 104 fewer to 0 fewer)
	372 (2 observational studies)	Very low	RR 0.76 (0.61 to 0.95)	544/1000	131 fewer per 1,00 (from 212 fewer to 27 fewer)
Survival to hospital discharge	591 (1 RCT)	Low	RR 1.04 (0.60 to 1.79)	80/1000	3 more per 1,000 (from 32 fewer to 63 more)
	4393 (5 propensity-matched observational)	Very low	RR 0.72 (0.48 to 1.07)	262/1000	73 fewer per 1,000 (from 136 fewer to 18 more)
	7392 (8 observational studies)	Very low	RR 0.85 (0.40 to 1.78)	196/1000	29 fewer per 1,000 (from 118 fewer to 153 more)
SGA (I) *vs* BMV (C)					
Survival with favourable neurologic outcome	3123 (4 propensity-matched observational)	Very low	RR 0.57 (0.26 to 1.23)	76/1000	33 fewer per 1,000 (from 56 fewer to 18 more)
Survival to hospital discharge	3122 (4 propensity-matched observational)	Very low	RR 0.89 (0.54 to 1.46)	126/1000	14 fewer per 1,000 (from 58 fewer to 58 more)
	3085 (2 observational studies)	Very low	RR 0.53 (0.21 to 1.34)	90/1000	43 fewer per 1,000 (from 71 fewer to 31 more)
TI (I) *vs* SGA (C)					
Survival with favourable neurologic outcome	1514 (3 propensity-matched observational)	Very low	RR 0.80 (0.44 to 1.43)	40/1000	8 fewer per 1,000 (from 23 fewer to 17 more)
	452 (1 observational studies)	Very low	RR 2.75 (0.67 to 11.27)	13/1000	24 more per 1,000 (from 4 fewer to 138 more)
Survival to hospital discharge	1514 (3 propensity-matched observational)	Very low	RR 0.80 (0.55 to 1.15)	126/1000	25 fewer per 1,000 (from 57 fewer to 19 more)
	1007 (3 observational studies)	Very low	RR 1.35 (0.82 to 2.22)	67/1000	24 more per 1,000 (from 12 fewer to 82 more)

**ARD:** absolute risk difference; **BMV**: bag-mask ventilation; **C**: comparator; **CI:** confidence interval; **GRADE**: Grading of Recommendations, Assessment, Development, and Evaluation; **I**: intervention; **RCT**: randomised controlled trial; **RR**: risk ratio; **SGA**: supraglottic airway; **TI**: tracheal intubation.

**Tracheal intubation (TI) compared to bag mask ventilation (BMV)**

#### Survival to hospital discharge with good neurologic outcome

We identified low certainty evidence downgraded for indirectness (resuscitations conducted prior to 2000, when standard resuscitation was different than current practice) and imprecision from 1 controlled clinical trial.^17^ This study assigned 591 children with out-of-hospital cardiac arrest (OHCA) to TI (even days) or BMV (odd days) and found no statistical benefit or harm associated with use of TI (RR 0.69, CI 95%: 0.32–1.52; absolute risk difference aRD: 15 fewer children surviving to hospital discharge with good neurologic outcome per 1,000 randomised to TI; CI 95%: 34 fewer to 26 more).

Additionally, very low certainty evidence comes from five propensity-adjusted cohort studies, downgraded for risk of bias because none could differentiate children with failed attempts at TI from those in whom TI was not attempted.^15^, ^18^, ^19^, ^20^, ^21^ These studies included 4,093 children with in-hospital cardiac arrest (n = 1970) or out-of-hospital cardiac arrest (n = 2123) and found no statistical benefit or harm associated with use of the TI intervention (RR 0.54, CI 95%: 0.29–1.00; aRD: 67 fewer children surviving to hospital discharge with good neurologic outcome per 1,000 resuscitations; CI 95%: 104 fewer to 0 fewer). These studies had heterogeneous results (I^2^: 73%). In addition, very low certainty evidence is available from 2 cohort studies involving 372 children (124 IHCA, 248 OHCA) which found apparent harm associated with the use of TI (RR 0.76, CI 95%: 0.61–0.95; aRD: 131 fewer children surviving to hospital discharge with good neurologic outcome per 1000 resuscitations; CI 95%: 212 fewer to 27 fewer).^26^, ^29^

Very low certainty evidence is available from 4 further observational studies with data not amenable to *meta*-analysis. Two studies found no statistical benefit or harm from advanced airway interventions (pooled TI/SGA but TI used in > 90% in each) compared to BMV (Fink^30^: adjusted odds ratio (aOR) 0.64, CI 95%: 0.37 – 1.13; Tijssen^14^: aOR 0.69, CI 95%: 0.43 – 1.10). One small cohort study^32^ reported a protective effect of advanced airway interventions (pooled TI/SGA − %TI unreported) compared to BMV for children surviving to hospital discharge with good neurologic outcome (OR 8.95, 95%CI 1.41–66.08) after OHCA.

#### Survival to hospital discharge

For this critical outcome, we identified low certainty evidence from one clinical trial of 591 children with OHCA.^17^ No apparent association was found between TI and survival (RR 1.04, CI 95%: 0.60–1.79; aRD: 3 more children surviving to hospital discharge per 1,000 assigned to TI; CI 95%: 32 fewer to 63 more).

Very low certainty evidence is provided by 5 propensity-adjusted cohort studies including 4,393 children.^15^, ^18^, ^19^, ^20^, ^21^ No apparent association was found between TI and survival (RR 0.72, CI 95%: 0.48–1.07; aRD: 73 fewer children surviving to hospital discharge per 1000 assigned to TI; CI 95%: 136 fewer to 18 more).

Combined data from 8 observational studies involving 7392 children were highly discordant (I^2^: 88%).^13^, ^23^, ^24^, ^25^, ^26^, ^27^, ^28^, ^29^ No apparent association was found between TI and survival to hospital discharge (RR 0.85, CI 95%: 0.40–1.78; aRD: 29 fewer children surviving to hospital discharge per 1000 assigned to TI; CI 95%: 118 fewer to 153 more).

**Supraglottic airway (SGA) compared to bag mask ventilation (BMV)**

No clinical trial studied the impact of SGA placement on resuscitation outcomes; the best available evidence for all outcomes is of very low certainty.

#### Survival to hospital discharge with good neurologic outcome

Very low certainty evidence obtained from 4 propensity-adjusted cohort studies involving 3,123 patients (all OHCA) showed no statistical benefit or harm associated with SGA ventilation (RR 0.57, CI 95%: 0.26–1.23; aRD: 33 fewer children surviving to hospital discharge with good neurologic outcome per 1,000 resuscitations; CI 95%: 56 fewer to 18 more).^15^, ^19^, ^20^, ^21^

#### Survival to hospital discharge

For this critical outcome, very low certainty evidence obtained from 4 propensity-adjusted cohort studies involving 3,123 children (all OHCA) showed no statistical benefit or harm associated with SGA ventilation (RR 0.89, CI 95%: 0.54–1.46; aRD: 14 fewer children surviving to hospital discharge per 1,000 resuscitations; CI 95%: 58 fewer to 58 more).^15^, ^19^, ^20^, ^21^

Additional very low certainty evidence from two observational studies of 3,085 children found no significant treatment association (RR 0.53, CI 95%: 0.21–1.34; aRD: 43 fewer children surviving to hospital discharge per 1000 treated with SGA; CI 95%: 71 fewer to 31 more).^23^, ^33^

**Tracheal intubation (TI) compared to supraglottic airway (SGA)**

No clinical trial studied the impact of SGA placement on resuscitation outcomes; the best available evidence is observational and of very low certainty.

#### Survival to hospital discharge with good neurologic outcome

For this critical outcome, very low certainty evidence is available from 3 propensity-matched cohort studies enrolling 1,514 children (all OHCA).^19^, ^20^, ^22^ When combined, these studies showed no statistical benefit or harm to either intervention (RR 0.80, CI 95%: 0.44–1.43; aRD: 8 fewer children surviving to hospital discharge with good neurologic outcome per 1,000 patients managed with TI rather than SGA; CI 95%: 23 fewer to 17 more). Additional very low certainty evidence is provided by a cohort study of 452 children with OHCA, which also found no statistical advantage to either modality (RR 2.75, CI 95%: 0.67–11.27; aRD: 24 more children surviving to hospital discharge with good neurologic outcome with TI; CI 95%: 4 fewer to 138 more).^15^

#### Survival to hospital discharge

Similar to the above, for this critical outcome, very low certainty evidence from 3 propensity-matched cohort studies of 1,514 children (all OHCA) found no statistical benefit or harm associated with TI or SGA (RR 0.80, CI 95%: 0.55–1.15; aRD: 25 fewer children surviving to hospital discharge per 1,000 patients managed with TI rather than SGA; CI 95%: 57 fewer to 19 more). ^19,20,22^ Similar results were obtained from 3 cohort studies involving 1007 children after OHCA (RR 1.35, CI 95%: 0.82–2.22; aRD: 24 more children surviving to hospital discharge per 1,000 patients managed with TI rather than SGA; CI 95%: 12 fewer to 82 more).^15^, ^23^, ^33^

**Subgroup analyses**

#### Out-of-hospital and in-hospital cardiac arrest

Separate analyses of studies of IHCA and OHCA produced similar results (see Supplemental Materials -S3**).** However, the body of evidence for IHCA is particularly small (consisting of 1 propensity-matched cohort study and 3 other cohort studies) and provides very low certainty evidence.^18^, ^26^, ^27^, ^34^ The studies are very heterogenous and showed inconsistent results.

#### Analyses by age group

One retrospective cohort study of 455 infants (age less than one year) provides very low certainty evidence about survival to hospital discharge following OHCA.^23^ This study reported no significant difference in survival to hospital discharge for children in cardiac arrest managed with TI compared with BMV (24 fewer children surviving to hospital discharge per 1000 managed with TI; CI: 61 fewer to 13 more); a significant survival disadvantage in patients managed with SGA compared with BMV (56 fewer children surviving to hospital discharge per 1000 managed with SGA; CI: 80 fewer to 32 fewer); and no significant difference in survival to hospital discharge when TI resuscitation was compared with SGA resuscitation (32 more children surviving to hospital discharge per 1000 resuscitations with TI compared with SGA; CI: 10 fewer to 73 more).

#### Subgroup analyses not conducted

No data could be found to support the preplanned subgroup analyses on the effect of AAW subdivided by initial rhythm (*shockable vs non-shockable),* type of arrest (*traumatic vs medical* or *primary respiratory arrest vs primary cardiac arrest*), or training of the person performing the AAW intervention (*paramedics vs physicians vs other professionals*).

## Discussion

Advanced airway interventions, particularly TI, have been long-established components of the advanced life support bundle of care in children. As a result of inherent limitations in their design and data sources, the available studies, though individually well conducted, can provide only very low certainty evidence about whether attempting advanced airway placement prior to ROSC improves resuscitation outcomes.

The best available data show no benefit from these advanced airway interventions for the critical outcomes of survival with good neurologic outcome and survival to hospital discharge. Our results suggest (with low to very low certainty) that resuscitation with tracheal intubation is not superior to BMV-based resuscitation for cardiac arrest in children. Based on limited and contradictory evidence of very low certainty, the overall data are also most consistent with no treatment effect associated with SGA ventilation when compared with BMV and no significant differences in outcomes shown between the use of TI or SGA in pediatric resuscitation.

Effective BMV, TI, and SGA are all difficult skills that require good initial training, retraining, and quality control to be done consistently, safely, and effectively. Paediatric advanced airway programs require a moderate investment in equipment and a significant investment in training, skills maintenance, and quality control programs to be successful.

The decision regarding the preferred choice of airway management technique (BMV, SGA or TI) in the setting of paediatric cardiac arrest is a complex one as the benefit or harm may differ across different settings, for different ages of children, for different causes of the arrest, and depending on the experience of the resuscitation team. Importantly, the available data do not inform the questions of whether better outcomes might be achieved by different airway strategies in long distance transportation or in prolonged resuscitation situations, with highly experienced airway operators. The analysed data are only relevant to advanced airway interventions during CPR, and do not pertain to airway management in other critical situations or once ROSC has been achieved.

### Limitations

Limitations to *meta*-analyses are well described. Mathematical combination of studies with differing designs, patient populations, and endpoint definitions is problematic, and small differences in analytic technique may change reported answers. Relative risk and risk difference calculations may produce divergent results when participant numbers are small (as was encountered in this review). Several of the studies contained significant methodological flaws including the inability to correctly classify patient with failed AAW attempts^18^, ^19^, ^20^ and exposure to AAW versus BMV-based resuscitation based on the availability of ALS-level resuscitation.^26^

Most of the available data has been obtained from registries and an unknown proportion of events labelled as BMV resuscitation may have had failed TI and/or SGA attempts (which would bias against BMV). Conversely, most of the included studies are susceptible to resuscitation-time bias i.e. the longer the child is in cardiac arrest, the more likely they will receive interventions but the less likely they will survive (which should bias against TI/SGA). Several factors that were not recorded (e.g. training or experience level of the clinician performing the airway management and ventilation) may, in reality, be more important than the choice of AAW intervention. In addition, the benefit or harm associated with AAW-based resuscitation is likely to differ between different resource settings.

#### Research priorities

Prehospital, ED-based, and in-hospital studies, ideally comparing TI, SGA and BMV with planned subgroup analyses based on patient age and aetiology of arrest (trauma *vs* non-trauma) are ethical, necessary, and critically important to help guide clinicians in making these complex decisions. Because cardiac arrests in children are rare, any such study would need to be multicentre and require high quality research infrastructure and data management facilities.

Further examination of the benefit of advanced airway interventions in particular settings (including patients with poor pulmonary compliance, long distance transportation) would be helpful. The efficacy and speed of placement of advanced airway using newer technologies, such as video assisted laryngoscopy (compared to regular laryngoscopy), is not known during resuscitation and would benefit from further studies.

Measures of the quality of chest compressions and ventilations were not consistently measured in the included studies. These may be important confounders in assessment of airway interventions and outcomes. Future studies would benefit from including measures of quality of ventilation and chest compressions, timing of airway intervention, duration of CPR and measures of the training and experience of the clinicians performing the interventions.

## Conclusions

There is currently no supporting evidence that an advanced airway (supraglottic airway or tracheal intubation) during CPR improves survival or survival with a good neurological outcome after paediatric cardiac arrest in any setting when compared with bag-mask ventilation. Results of this systematic review were used to inform the 2024 International Consensus on Cardiopulmonary Resuscitation and Emergency Cardiovascular Care Science With Treatment Recommendations.^35^

## CRediT authorship contribution statement

**Jason Acworth:** Writing – review & editing, Writing – original draft, Validation, Supervision, Project administration, Methodology, Formal analysis, Data curation, Conceptualization. **Jimena del Castillo:** Writing – review & editing, Formal analysis, Data curation. **Elliott Acworth:** Writing – review & editing, Validation, Data curation. **Lokesh Tiwari:** Writing – review & editing, Data curation. **Jesus Lopez-Herce:** Writing – review & editing, Data curation. **Eric Lavonas:** Writing – review & editing, Methodology, Conceptualization. **Laurie Morrison:** Writing – review & editing, Methodology. **Barnaby R. Scholefield:** Writing – review & editing, Supervision, Project administration.

## Funding

This research did not receive any specific grant from funding agencies in the public, commercial, or not-for-profit sectors. This Systematic Review was conducted through volunteer support from the International Liaison Committee On Resuscitation (ILCOR) which receives funding from the American Heart Association.

## Declaration of competing interest

The authors declare that they have no known competing financial interests or personal relationships that could have appeared to influence the work reported in this paper.

Barnaby R Scholefield serves on the Editorial Board of the Resuscitation Journal. He was not involved in the editorial review or the decision to publish this article.

## Appendix

**Non-Author Contributors:**

The authors would like to formally acknowledge the following persons as contributors:

University of Queensland Library (Information Specialist):

1. Lars Eriksson: l.eriksson@library.uq.edu.au

ILCOR Paediatric Life Support Task Force:

1. Ester Ambunda: esterambunda@gmail.com
2. Allan DeCaen: allan.decaen@albertahealthservices.ca
3. Dianne Atkins: Dianne-atkins@uiowa.edu
4. Arun Bansal: drarunbansal@gmail.com
5. Thomaz Bittencourt-Cuoto: thomaz.couto@hc.fm.usp.br
6. Andrea Christoff: andrea.christoff@health.nsw.gov.au
7. Jana Djakow: jana.djakow@gmail.com
8. Raffo Escalante-Kanashiro: raffo.escalante@gmail.com
9. James M Gray: James.Gray@cchmc.org
10. Anne-Marie Guergerian: anne-marie.guerguerian@sickkids.ca
11. Stephan Katzenschlager: Stephan.Katzenschlager@med.uni-heidelberg.de
12. Monica Kleinman: monica.kleinman@childrens.harvard.edu
13. Hiroshi Kurosawa: kurohiro2@gmail.com
14. Michelle Myburgh: Myburghmc@ufs.ac.za
15. Kee-Chong Ng: Ng.kee.chong@kkh.com.sg
16. Vinay Nadkarni: nadkarni@email.chop.edu
17. Gabrielle Nuthall: gabriellen@adhb.govt.nz
18. Yong-Kwang Gene Ong: geneong@yahoo.com
19. Tia T Raymond: tiaraymond@me.com
20. Joseph Rossano: rossanoj@chop.edu
21. Steve Schexnayder: schexnaydersm@uams.edu
22. Janice Tijssen: janice.tijssen@lhsc.on.ca
23. Alexis Topjian: topjian@chop.edu
