# Supplementary material for: Advanced airway interventions for paediatric cardiac arrest: updated systematic review and meta-analysis
Source: Resusc Plus. 2025 Apr 23;23:100963. doi: 10.1016/j.resplu.2025.100963 (PMC12138473; doi:10.1016/j.resplu.2025.100963)
Supplement: Supplementary Data 2 [file mmc2.docx]

**Supplement 2: Search Strategy for Advanced airway interventions for paediatric cardiac arrest - updated systematic review and meta-analysis**

**PUBMED:**

(((("supraglottic"[Title/Abstract] OR "laryngeal mask"[Title/Abstract] OR "LMA"[Title/Abstract] OR "combitube"[Title/Abstract] OR "EasyTube"[Title/Abstract] OR "king airway"[Title/Abstract] OR "I-Gel"[Title/Abstract] OR "ProSeal"[Title/Abstract] OR "CTrach"[Title/Abstract] OR "esophageal obturator"[Title/Abstract] OR "bag-mask"[Title/Abstract] OR "bag-valve-mask"[Title/Abstract] OR "bag-mask"[Title/Abstract] OR "bag-valve-mask"[Title/Abstract] OR "advanced airway"[Title/Abstract] OR ("intubation"[Title/Abstract] OR "intratracheal tube"[Title/Abstract] OR "endotracheal tube"[Title/Abstract] OR "ETT"[Title/Abstract])) AND ("infan*"[Text Word] OR "child*"[Text Word] OR "adolescen*"[Text Word] OR "pediatric*"[Text Word] OR "paediatric*"[Text Word] OR "pube*"[Text Word] OR "juvenil*"[Text Word] OR "school*"[Text Word] OR "newborn*"[Title/Abstract] OR "newborn*"[Title/Abstract] OR "neonat*"[Title/Abstract] OR "neonat*"[Title/Abstract] OR "premature*"[Title/Abstract] OR "postmature*"[Title/Abstract] OR "premature*"[Title/Abstract] OR "post mature*"[Title/Abstract] OR "preterm*"[Title/Abstract] OR "preterm*"[Title/Abstract] OR "baby"[Title/Abstract] OR "babies"[Title/Abstract] OR "toddler*"[Title/Abstract] OR "youngster*"[Title/Abstract] OR "preschool*"[Title/Abstract] OR "kindergart*"[Title/Abstract] OR "kid"[Title/Abstract] OR "kids"[Title/Abstract] OR "playgroup*"[Title/Abstract] OR "play group*"[Title/Abstract] OR "playschool*"[Title/Abstract] OR "prepube*"[Title/Abstract] OR "preadolescen*"[Title/Abstract] OR "junior high*"[Title/Abstract] OR "highschool*"[Title/Abstract] OR "senior high"[Title/Abstract] OR "young people*"[Title/Abstract] OR "minors"[Title/Abstract]) AND ("life support care"[MeSH Terms] OR "life support"[Title/Abstract] OR "cardiopulmonary resuscitation"[MeSH Terms] OR "cardiopulmonary resuscitation"[Title/Abstract] OR "ROSC"[Title/Abstract] OR "return of spontaneous circulation"[Title/Abstract] OR "heart arrest"[MeSH Terms] OR "cardiac arrest"[Title/Abstract])) NOT ("animals"[MeSH Terms] NOT "humans"[MeSH Terms])) NOT ("Letter"[Publication Type] OR "Editorial"[Publication Type] OR "Comment"[Publication Type])) AND 2018/07/01:2023/12/31[Date - Publication]

**EMBASE:**

('intubation' OR 'respiratory tract intubation' OR 'endotracheal intubation' OR 'assisted ventilation' OR 'artificial ventilation' OR 'laryngeal mask' OR 'respiration control' OR ((rescue OR emergency) NEXT/1 intubation*) OR ((intubation* NEXT/2 trachea):ab,kw,ti) OR 'supraglottic airway device' OR supraglottic:ab,kw,ti OR (((endotracheal OR intratracheal OR tracheal) NEXT/2 (intubation* OR tube*)):ab,kw,ti) OR ((laryngeal NEXT/1 (mask* OR tube*)):ab,kw,ti) OR combitube:ab,kw,ti OR ((esophageal NEXT/2 trachea* NEXT/2 (tube* OR airway)):ab,kw,ti) OR 'king airway':ab,kw,ti OR 'i-gel':ab,kw,ti OR 'proseal':ab,kw,ti OR 'ctrach':ab,kw,ti OR 'guardiancpv':ab,kw,ti OR 'cobrapla':ab,kw,ti OR 'slipa':ab,kw,ti OR 'aura-i':ab,kw,ti OR 'esophagus obturator' OR 'esophageal obturator':ab,kw,ti OR 'esophageal gastric tube*':ab,kw,ti OR 'manual emergency ventilator' OR 'bag-mask':ab,kw,ti OR 'bag-valve-mask':ab,kw,ti OR 'advanced airway':ab,kw,ti OR ((intubation NEXT/2 (direct OR video OR fiberoptic) NEXT/2 laryngoscopy):ab,kw,ti)) AND ('heart arrest' OR 'cardiopulmonary arrest' OR 'out of hospital cardiac arrest' OR 'sudden cardiac death' OR 'resuscitation' OR 'advanced cardiac life support':ab,kw,ti OR (((heart OR cardiac OR cardiovascular) NEXT/1 arrest):ab,kw,ti) OR 'return of spontaneous circulation' OR acls:ab,kw,ti OR (((cardiopulmonary OR 'cardio pulmonary') NEXT/1 (arrest OR resuscitation)):ab,kw,ti) OR cpr:ab,kw,ti OR 'chest compressions':ab,kw,ti OR reanimation:ab,kw,ti OR 'respiratory arrest' OR 'respiratory arrest':ab,kw,ti OR (('cardiac arrest' NEXT/2 registr*):ab,kw,ti) OR 'emergency' OR 'emergency medicine' OR 'pediatric emergency medicine' OR 'emergency health service' OR 'hospital emergency service' OR emergenc*:ab,ti,kw) AND ('child' OR 'adolescent' OR 'infant' OR 'pediatrics' OR (((infan*:ab,kw,ti OR baby:ab,kw,ti OR baby*:ab,kw,ti OR babies:ab,kw,ti OR toddler*:ab,kw,ti OR boy:ab,kw,ti OR boys:ab,kw,ti OR boyhood:ab,kw,ti OR girl*:ab,kw,ti OR kid:ab,kw,ti OR kids:ab,kw,ti OR child:ab,kw,ti OR child*:ab,kw,ti OR children*:ab,kw,ti OR schoolchild*:ab,kw,ti OR schoolchild:ab,kw,ti OR school:ab,kw,ti) AND child:ab,kw,ti OR school:ab,kw,ti) AND child*:ab,kw,ti) OR adolescen*:ab,kw,ti OR juvenil*:ab,kw,ti OR youth*:ab,kw,ti OR teen*:ab,kw,ti OR under*age*:ab,kw,ti OR pubescen*:ab,kw,ti OR pediatric*:ab,kw,ti OR paediatric*:ab,kw,ti OR peadiatric*:ab,kw,ti) NOT (animal NOT human) NOT ([conference abstract]/lim OR [conference paper]/lim OR [conference review]/lim OR [editorial]/lim OR 'case report') AND ([newborn]/lim OR [infant]/lim OR [child]/lim OR [preschool]/lim OR [school]/lim OR [adolescent]/lim) AND [01-07-2018]/sd NOT [01-01-2024]/sd

**COCHRANE:**

ID Search

#1 MeSH descriptor: [Intubation] this term only

#2 MeSH descriptor: [Intubation, Intratracheal] explode all trees

#3 MeSH descriptor: [Respiration, Artificial] this term only

#4 MeSH descriptor: [Airway Management] this term only

#5 ((endotracheal or Intratracheal or tracheal) near/1 (intubation* or tube*)):ti,ab,kw

#6 ((rescue or emergency) near/1 intubation*):ti,ab,kw

#7 (intubation near/2 trachea):ti,ab,kw

#8 (Supraglottic):ti,ab,kw

#9 (Laryngeal near/1 (Mask* or tube*)):ti,ab,kw

#10 Combitube or EasyTube

#11 ((esophageal) near/2 (trachea*) near/2 (tube* or airway)):ti,ab,kw

#12 "king airway" or I-Gel or ProSeal or CTrach or bag-mask or bag-valve-mask or "advanced airway"

#13 "esophageal obturator" or "esophageal gastric tube"

#14 ((intubation) near/2 (direct or video or fiberoptic) near/2 (laryngoscopy)):ti,ab,kw

#15 #1or#2or#3or#4or#5or#6or#7or#8or#9or#10or#11or#12or#13or#14

#16 MeSH descriptor: [Heart Arrest] explode all trees

#17 MeSH descriptor: [Cardiopulmonary Resuscitation] explode all trees

#18 ((heart or cardiac or cardiovascular) near/1 arrest):ti,ab,kw

#19 "return of spontaneous circulation"

#20 ACLS

#21 ((cardiopulmonary or cardio-pulmonary) near/1 (arrest or resuscitation)):ti,ab,kw

#22 CPR or "chest compressions" or reanimation

#23 MeSH descriptor: [Resuscitation] this term only

#24 "respiratory arrest"

#25 ((Cardiac Arrest) near/2 (Registr*)):ti,ab,kw

#26 (emergenc*.):ti,ab,kw

#27 MeSH descriptor: [Emergency Medical Services] this term only

#28 MeSH descriptor: [Emergency Medicine] explode all trees

#29 MeSH descriptor: [Emergency Service, Hospital] explode all trees

#30 #16or#17or#18or#19or#20or#21or#22or#23or#24or#25or#26or#27or#28or#29

#31 MeSH descriptor: [Adolescent] explode all trees

#32 MeSH descriptor: [Child] explode all trees

#33 MeSH descriptor: [Infant] explode all trees

#34 MeSH descriptor: [Pediatrics] this term only

#35 (baby or babies or toddler or kid or child or children or schoolchild or "school child" or adolescen* or juvenil or youth or teen or underage or pubescen or pediatric or paediatric or peadiatric):ti,ab,kw

#36 #31 or #32 or #33 or #34 or #35

#37 #15 and #30 and #36

#38 #37 with Cochrane Library publication date Between Jul 2018 and Dec 2023
