# Supplementary material for: Advanced airway interventions for paediatric cardiac arrest: updated systematic review and meta-analysis
Source: Resusc Plus. 2025 Apr 23;23:100963. doi: 10.1016/j.resplu.2025.100963 (PMC12138473; doi:10.1016/j.resplu.2025.100963)
Supplement: Supplementary Data 3 [file mmc3.docx]

**Supplement 3: Risk of Bias assessment**

| Clinical Trials | | | | | | | | | |
| --- | --- | --- | --- | --- | --- | --- | --- | --- | --- |
| GRADE Risk of Bias Elements for Clinical Trials | Random sequence generation | Allocation concealment | Blinding of participants and personnel | Blinding of outcome assessment | Incomplete outcome data | Selective reporting | Other bias | | Overall risk of bias |
| Gausche 2000 | Low Risk (1) | Low Risk | Low Risk (2) | Low Risk (2) | Low Risk | Low Risk | Uncertain risk (3, 4, 5, 6) | | Not Serious |
| Cohort Studies | | | | | | | | | |
| CLARITY Risk of Bias Elements for Cohort Studies | Was selection of exposed and non-exposed cohorts drawn from the same population? | Can we be confident in the assessment of exposure? | Can we be confident that the outcome of interest was not present at start of study? | Did the study match exposed & unexposed for all variables associated with outcome of interest or did statistical analysis adjust for these prognostic variables? | Can we be confident in the assessment of the presence or absence of prognostic factors? | Can we be confident in the assessment of outcome? | Was the follow up of cohorts adequate? | Were co-interventions similar between groups? | Overall risk of bias |
| Cohort Studies - Propensity Matched | | | | | | | | | |
| Andersen 2016 | Definitely yes | Definitely no (7) | Definitely yes | Probably yes (8) | Probably yes | Definitely yes | Probably yes | Probably no (9) | Serious |
| Hansen 2017 | Definitely yes | Definitely no (7, 10) | Definitely yes | Probably yes (8) | Probably yes | Definitely yes | Probably yes | Probably no (9) | Serious |
| Ohashi-Fukuda 2017 | Definitely yes | Definitely no (7, 10) | Definitely yes | Probably yes (8) | Probably yes | Definitely yes | Probably yes | Probably no (9) | Serious |
| Okubo 2019 | Definitely yes | Definitely no (7, 10) | Definitely yes | Probably yes (8) | Probably yes | Definitely yes | Probably yes | Probably no (9) | Serious |
| Fukuda 2020 | Definitely yes | Definitely no (7, 10) | Definitely yes | Probably yes (8) | Probably yes | Definitely yes | Probably yes | Probably no (9) | Serious |
| Tham 2022 | Definitely yes | Definitely no (7, 10) | Definitely yes | Probably yes (8) | Probably yes | Definitely yes | Probably yes | Probably no (9) | Serious |
| Cohort Studies - Not Propensity Matched | | | | | | | | | |
| Abe 2012 | Definitely yes | Probably yes (10) | Definitely yes | Definitely no (11) | Definitely no | Definitely yes | Probably yes | Probably no (9) | Very serious |
| Aijian 1989 | Definitely yes | Probably yes (9) | Definitely yes | Definitely no (11) | Definitely no | Definitely yes | Probably yes | Probably no (9) | Very serious |
| Deasy 2010 | Definitely yes | Definitely no (7, 10) | Definitely yes | Definitely no (11) | Definitely no | Definitely yes | Probably yes | Probably no (9) | Very serious |
| del Castillo 2015 | Definitely yes | Definitely no (7) | Definitely yes | Definitely no (11) | Definitely no | Definitely yes | Probably yes | Probably no (9) | Very serious |
| Guay 2004 | Definitely yes | Definitely no (7) | Definitely yes | Definitely no (11) | Definitely no | Definitely yes | Probably yes | Probably no (9) | Very serious |
| Pitetti 2002 | Definitely yes | Probably no (10, 12) | Definitely yes | Definitely no (11) | Definitely no | Definitely yes | Probably yes | Definitely no (13) | Very serious |
| Sirbaugh 1999 | Definitely yes | Definitely no (7, 10) | Definitely yes | Definitely no (11) | Definitely no | Definitely yes | Probably yes | Probably no (9) | Very serious |
| Hansen 2020 | Definitely yes | Definitely no (10) | Definitely yes | Definitely no (11) | Definitely no | Definitely yes | Probably yes | Probably no (9) | Very serious |
| Handley 2021 | Definitely yes | Definitely no (7) | Definitely yes | Definitely no (11) | Definitely no | Definitely yes | Probably yes | Probably no (9) | Very serious |
| Cohort Studies - Not Propensity Matched, Not Amenable to Meta-Analysis | | | | | | | | | |
| Fink 2016 | Definitely yes | Probably yes (10, 14) | Definitely yes | Definitely no (11) | Definitely no | Definitely yes | Probably yes | Probably yes (15) | Very serious |
| Tijssen 2014 | Definitely yes | Probably yes (10, 14) | Definitely yes | Definitely no (11) | Definitely no | Definitely yes | Probably yes | Probably yes (15) | Very serious |
| LeBastard 2021 | Definitely yes | Probably yes (10) | Definitely yes | Probably yes (8) | Probably yes | Definitely yes | Probably yes | Definitely no (16) | Serious |
| Cheng 2021 | Definitely yes | Probably yes (10, 17) | Definitely yes | Definitely no (11) | Definitely no | Definitely yes | Probably yes | Probably yes (15) | Very serious |

**Notes:**

1. Odd-even day pseudo-randomisation may introduce a small amount of bias
2. Although blinding is impossible, the use of objective endpoints, such as survival, mitigates this risk of bias
3. Indirectness: Significant changes have been made in standard resuscitation care since this study was conducted
4. Indirectness: Study was conducted around the introduction of paediatric intubation into the EMS system (therefore all paramedics newly trained, therefore inexperienced but with less opportunity for skill atrophy
5. Intention to treat analysis with moderate crossover (2% of patients randomised to BVM received TI prehospital, and unknown number had TI attempts; 27% of patients randomised to TI did not have TI attempted prehospital)
6. Unknown number of patients who were not intubated prehospital were subsequently intubated in the ED, potentially diluting the effect of prehospital airway management
7. Study is unable to distinguish patients with unsuccessful advanced airway attempts from those with no advanced airway attempt
8. Propensity matching methods were rigorous, but residual uncertainty about the effectiveness of propensity adjustment always exists
9. No clear presentation of co-interventions received in the AAW and non-AAW groups
10. Unknown number of patients who were not intubated prehospital received TI in the emergency department, thereby diluting the benefit / harm associated with prehospital AAW
11. Unclear how subjects were chosen to receive or not receive AAW or BVM alone; very likely the AAW / no AAW groups differed systematically in important ways
12. All ALS patients assumed to have TI attempted (92% were successfully intubated)
13. Patients in the AAW group also received other ALS interventions not available to no AAW patients, who received BLS resuscitation only
14. AAW group combined patients with TI and SGA; more than 90% of subjects received the same intervention (TI)
15. Adjusted odds ratio calculations attempted to control for effect of co-interventions, but specific formula not provided and therefore effectiveness of these controls unclear
16. Statistically significant differences in Bystander compression only CPR, BLS by first response team, Defibrillation, IO/IV access, Adrenaline administration. Tendency to more intervention in the ETI group.
17. AAW group combined patients with TI and SGA, nil clarification on proportion
