# Supplementary material for: Advanced airway interventions for paediatric cardiac arrest: updated systematic review and meta-analysis
Source: Resusc Plus. 2025 Apr 23;23:100963. doi: 10.1016/j.resplu.2025.100963 (PMC12138473; doi:10.1016/j.resplu.2025.100963)
Supplement: Supplementary Data 4 [file mmc4.docx]

**Supplement 4:** Forest Plots comparing airway intervention for infants and children for the critical outcomes of Survival with Good Neurologic Function and Survival to Hospital Discharge for subgroups OHCA and IHCA

1. **TI vs BMV in OHCA: Survival with Good Neurologic Function**


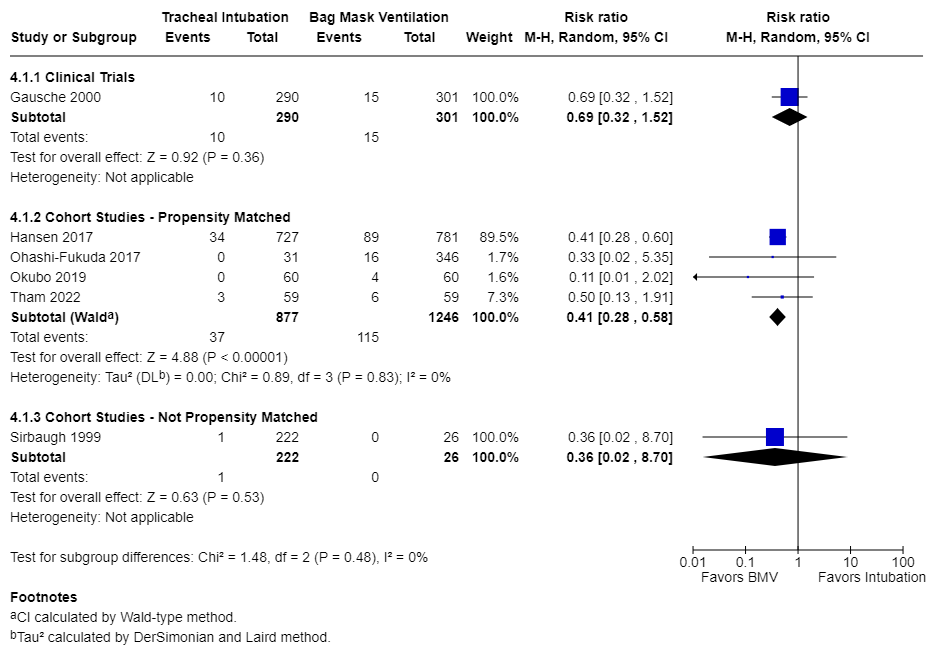


1. **TI vs BMV in OHCA: Survival to Hospital Discharge**

**
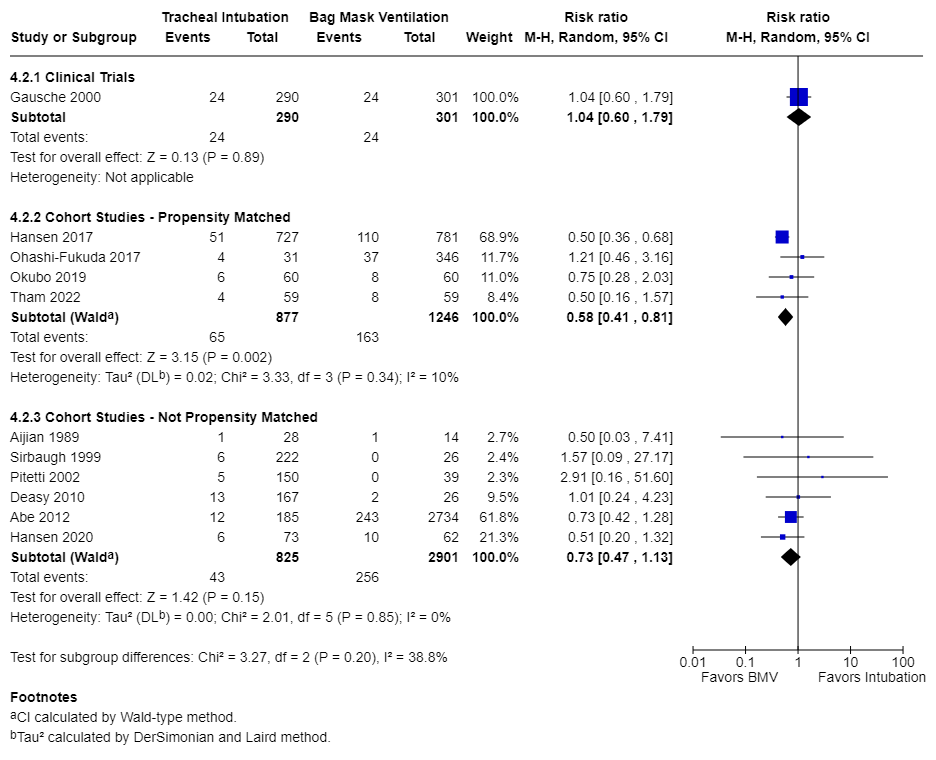
**

**M-H**: Cochran-Mantel-Haenszel. **95% CI**: 95 percent confidence interval

1. **SGA vs BMV in OHCA: Survival with Good Neurologic Function**


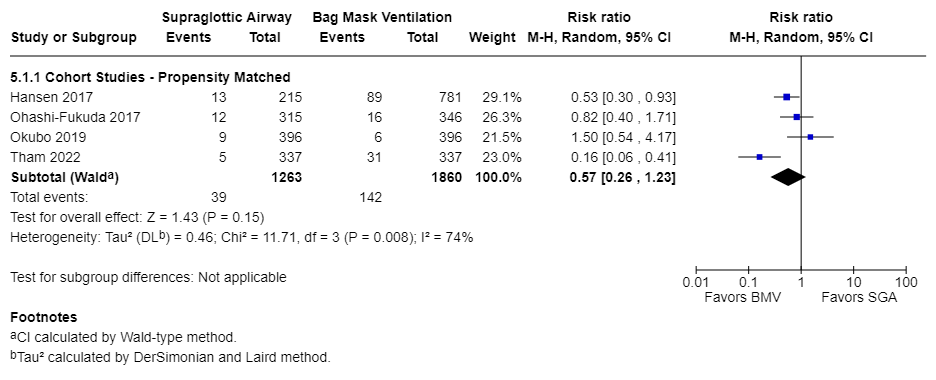


1. **SGA vs BMV in OHCA: Survival to Hospital Discharge**


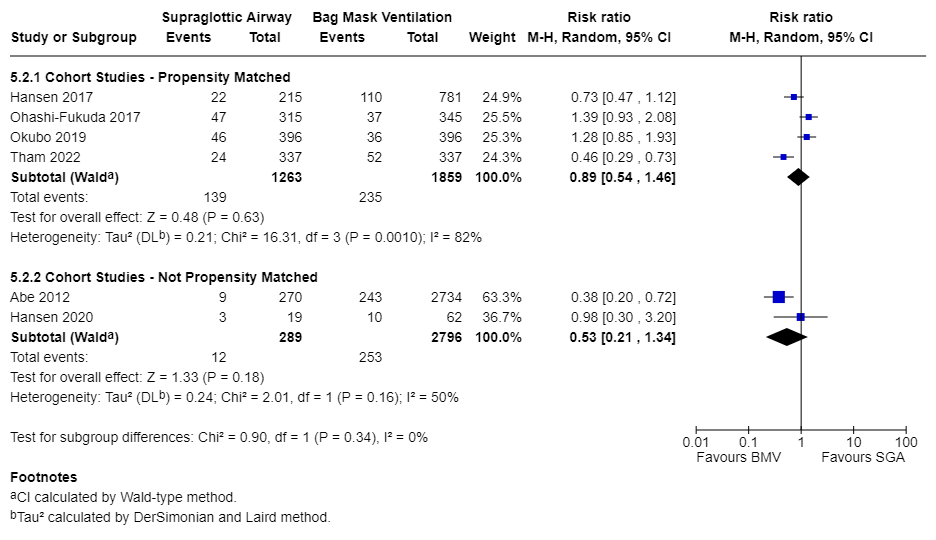


**M-H**: Cochran-Mantel-Haenszel. **95% CI**: 95 percent confidence interval

1. **TI vs SGA in OHCA: Survival with Good Neurologic Function**


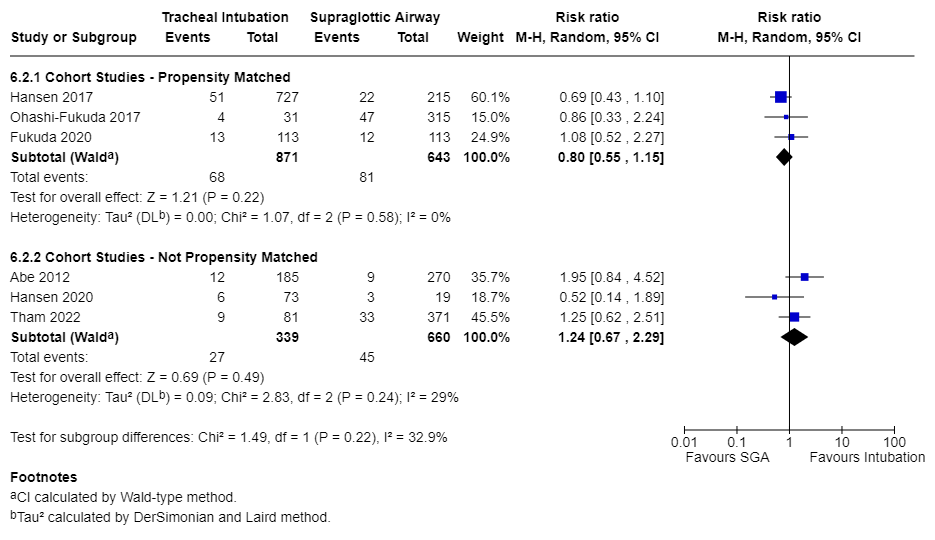


1. **TI vs SGA in OHCA: Survival to Hospital Discharge**


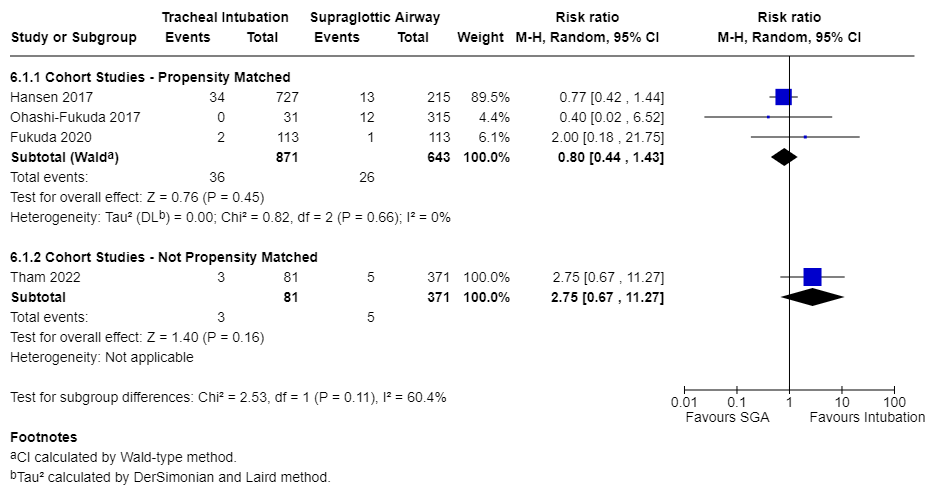


**M-H**: Cochran-Mantel-Haenszel. **95% CI**: 95 percent confidence interval

1. **TI vs BMV in IHCA: Survival with Good Neurologic Function**

**
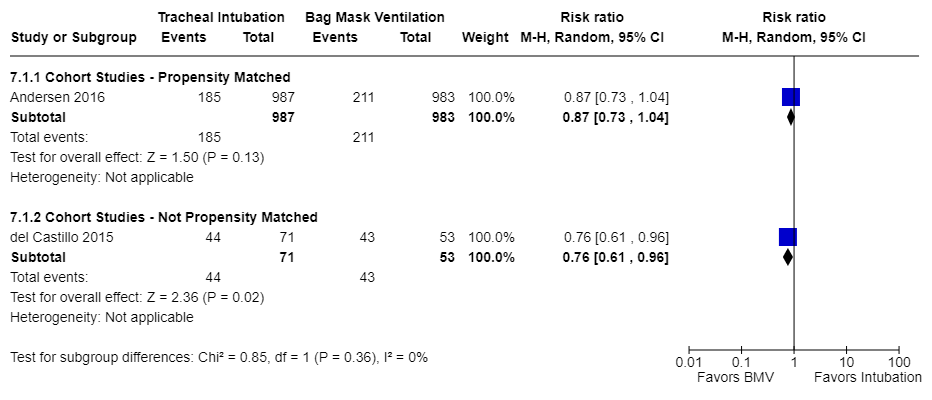
**

1. **TI vs BMV in IHCA: Survival to Hospital Discharge**

**
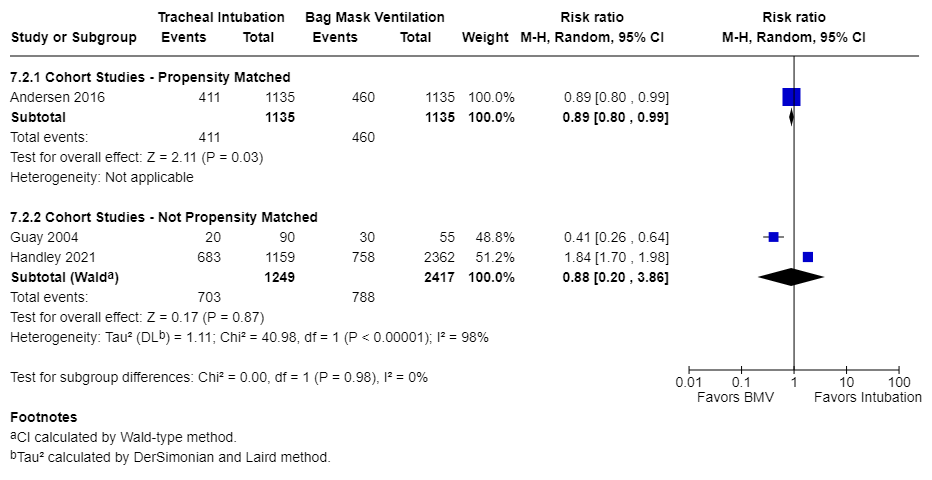
**

**M-H**: Cochran-Mantel-Haenszel. **95% CI**: 95 percent confidence interval
